# The development of cultural competences in nursing students and their significance in shaping the future work environment: a pilot study

**DOI:** 10.1186/s12909-023-04800-5

**Published:** 2023-11-01

**Authors:** Sabina Ličen, Mirko Prosen

**Affiliations:** Department of Nursing, Faculty of Health Sciences, University of Primorska, Polje 42, 6310 Izola, Slovenia

**Keywords:** Cultural competence, Cultural competence Assessment Tool, Nursing students, Transcultural nursing, Nursing curriculum

## Abstract

**Background:**

Working in a culturally diverse environment entails a moral and professional responsibility to provide culturally competent care. This has been recognised as an important measure to reduce health inequalities, improve the quality of care and increase patient satisfaction. The aim of this study was to assess the level of cultural competence in nursing students a decade after the introduction of transcultural nursing content into the nursing curriculum in Slovenia.

**Methods:**

A descriptive cross-sectional design with 180 s-year nursing students as a convenience sample was used. Cultural competence was assessed using the Cultural Competence Assessment Tool (CCATool) via an online survey. IBM SPSS facilitated statistical analysis, using descriptive statistics and inferential methods, including the chi-square test. Non-parametric tests (Mann Whitney U, Kruskal-Wallis H and Wilcoxon signed-rank) were used for non-normally distributed data (Shapiro-Wilk test, p < 0.05). The significance was set at p ≤ 0.05.

**Results:**

The results indicate that students demonstrate a high level of cultural competence, although there is room for improvement in terms of cultural sensitivity, as determined by coding the CCATool. The results also show a remarkable contrast between their self-assessed scores and the objective scores obtained from CCATool coding of the statements in each subscale (p < 0.005). In addition, significant differences (p = 0.002) are found in subscale “Cultural Knowledge” particularly between students who have lived abroad for more than 6 months and those who have not. The latter group has a higher score in the CCATool, indicating greater cultural knowledge.

**Conclusions:**

The study suggests that the presence of transcultural elements in the Slovenian nursing curriculum is associated with higher self-reported levels of cultural competence among nursing students, although the present research design does not allow for causal interpretations. This competence is of immense importance in preparing students for their future professional environment. However, it is crucial to further refine the nursing curriculum, especially through greater integration of transcultural content in all health disciplines. In addition, the introduction of innovative teaching and learning approaches can better prepare students to deal with the diverse cultural experiences they will encounter in their nursing careers.

## Introduction

Globalisation processes, epidemiological conditions, social conflicts and natural disasters often lead to increased migration flows. According to the International Organization for Migration [1], there were approximately 281 million international migrants worldwide in 2020, representing 3.6 per cent of the world’s population. This number would most likely have increased to 283 million had it not been for COVID-19. Europe is currently the largest destination for international migrants, with a total of 87 million migrants (30.9% of the international migrant population). Due to the war in Ukraine, these numbers are currently increasing rapidly. The resulting increase in cultural diversity is a challenge for all social structures in any host country, and also has an impact on the health system, especially on healthcare providers, who are obliged to provide quality care to patients of all cultural backgrounds [2]. Working in a culturally diverse environment places a moral and professional responsibility on all nursing students, nurses, their work organisations, educational institutions, professional bodies and the health system to provide culturally competent care [3, 4]. While this has been recognised as an important measure to reduce health inequalities, improve quality of care and increase patient satisfaction, it also means that future nurses need to be adequately prepared, and that the process of education and socialisation should begin at undergraduate level, where the values and norms of the nursing profession are fundamentally shaped [3, 5].

### Background

Nurses not only represent the largest group of healthcare professionals in the health system, but also spend most of their working time providing direct care to patients [2]. This means that they need to understand patients in the context of their cultural background. Sufficient knowledge about different cultures and traditions is also necessary to perform a comprehensive patient assessment [6]. Cultural competence has been at the centre of the debate in nursing for over half a century and is now an essential element of nursing practice, as well as an indicator of the level of professionalism in nursing. While some argue that the concept of cultural competence lacks clarity [7], the commonalities in the foundations of this concept remain the same despite certain problems with nomenclature. Cultural competence can be defined as the ability to provide culturally sensitive and competent care to patients and their families or communities with, taking into account their values, beliefs, customs and traditions, and incorporating them in the provision of care [8]. Cultural competence is the ability to provide culturally sensitive and competent care to patients, families or communities by considering their values, beliefs, customs and traditions and incorporating them in the provision of care [8]. According to Papadopoulos, Tilki and Taylor’s 1998 model, cultural competence involves providing effective healthcare while taking into account individuals’ cultural beliefs, behaviours and needs. It is seen as a continuous developmental process resulting from the integration of knowledge and skills acquired throughout one’s personal and professional life [9]. It is both a dynamic process and its outcome, which enables the provision of effective, safe, unbiased and quality care [4, 10]. A culturally competent healthcare professional is one who possesses the virtue of courage to challenge accepted norms, practices and even values that may harm, discriminate against or disadvantage health service users in any way [10].

The cultural competence of student nurses is of paramount importance in providing effective and compassionate care to diverse patient populations. Through education and exposure to different cultures, they develop a deep understanding and appreciation of the beliefs, values and practises of others. This enables them to provide patient-centred care, build trusting relationships and manage intercultural communication problems. By promoting cultural competence, nursing students contribute to more inclusive and equitable healthcare that respects and meets the unique needs of each individual [11, 12].

The two dimensions within the concept of cultural competence – ‘sensitivity’ and ‘culture’ – can be studied together, as cultural sensibility represents an openness to emotional impressions, receptivity, and sensitivity, which allows for personal reflection and influences human behaviour when interacting with others [3]. As explained by the authors, addressing cultural sensibility in nursing curricula should represent the first step in helping students understand the importance of culture and its role in caring for patients from different cultures. To facilitate training in cultural sensibility and to support the development of cultural competence in educational settings, it is essential to assess students’ level of understanding and cultural awareness [3, 5].

Cultural sensitivity and cultural awareness are not the only attributes of cultural competence. Cultural skills, cultural knowledge, cultural proficiency, and dynamism are all concepts underlying cultural competence [4, 13]. Although the concept of cultural proficiency may seem less prominent in discussions of cultural competence, it is nevertheless a key component of clinical nursing and a cornerstone for the advancement of transcultural nursing practice. Cultural proficiency refers to the acquisition and research-based knowledge transfer with therapeutic approaches. It involves a proactive approach and reflects a commitment to change. Its focus is on behaviour, not emotions, and it can be applied to organisational practices as well as individual behaviours [4, 14]. Cultural proficiency is a transformational model and as such also provides teachers and designers of study programmes with important feedback on their success in student transformation during their education.

Over the past two decades, several new concepts have emerged in the discipline of transcultural nursing which present cultural competence in a new light. One of these concepts is culturally competent compassion. Papadopoulos [10] believes that culturally competent nursing is undoubtedly compassionate nursing. She defines it as a human quality of understanding the suffering of others and helping them in a culturally appropriate and acceptable manner, which involves taking into consideration the cultural background of both patients and carers, as well as the context of care. Another, perhaps more challenging concept is cultural humility. It is defined as characteristic of respect, empathy, and self-reflection/self-criticism at the intrapersonal and interpersonal levels. At the intrapersonal level, it involves an awareness of one’s limited ability to understand the patient’s worldview and cultural characteristics, while at the interpersonal level, it involves an attitude of openness and respect towards the patient’s worldview [15]. Despite these different perspectives, both cultural competence and cultural humility are effectively used to encourage self-reflection and promote reflective practice in relation to patients’ cultural characteristics. Both concepts also emphasise the need to challenge the institutions and systems that allow for injustices which lead to inequalities, and to show that we can work together in this endeavour [16].

Curriculum interventions have been shown to contribute to the development of cultural competence in nursing students [17–19]; however, in Slovenia such interventions were rarely introduced before 2013 [5]. Undergraduate nursing education in Slovenia is in line with the Directive on the Recognition of Professional Qualifications 2005/36/ES and 2013/55/EU as well as the guidelines of the Bologna Declaration. In Slovenia, at the University of Primorska, Faculty of Health Sciences (UP FHS), the process of changing the nursing curriculum by introducing the topic of transcultural nursing began in 2013 with the development of a 25-hour elective course ‘Transcultural Nursing’ at the postgraduate level [5], which was changed in 2020 to a mandatory 90-hour course entitled ‘Transcultural Nursing and Global Health’. In 2014, an additional lecture on transcultural nursing was added to the second-year undergraduate nursing curriculum as part of the mandatory course ‘Sociology of Health and Illness’. Ever since, cultural competence has been widely promoted among both students and student mentors in the clinical setting. The aim of this study was to assess level of cultural competence of nursing students’ a decade after the introduction of transcultural nursing content in the nursing curriculum in Slovenia.

## Method

### Research design

To achieve the aim of the study, a cross-sectional study was conducted on a convenience sample of undergraduate nursing students.

### Research questions

The purpose of the study is to gain a comprehensive understanding of the characteristics and experiences of the participants, thus improving the validity and applicability of the study’s findings. By collecting baseline data on students’ previous experiences with cultural diversity and exposure to transcultural care concepts, the study will provide a solid foundation for future comparisons between students with varying levels of cultural knowledge. Understanding students’ cultural and demographic backgrounds is critical to interpreting study findings and offers insights into how variables, such as cultural experiences and personal background, define their level of cultural competence.

The research questions are as follows: (a) To what extent do student nurses demonstrate cultural competence? (b) What are the discrepancies between self-assessed scores and objective scores obtained by coding statements on each subscale in CCATool? and (c) How do demographic and other social characteristics of student nurses define their level of cultural competence and what differences are observed between them?

### Participants

The sample was formed from 317 s-year undergraduate nursing students in the academic year 2021/2022 at one of the nursing faculties in Slovenia. Invitations to participate in the survey were sent to students through the Office of Student Affairs, along with a detailed explanation of the purpose of the survey. The calculated sample size, based on a confidence level of 95% with a margin of error of 5%, was 174, which means that we achieve representativeness of the sample of nursing students (n = 180) among whom the survey was conducted.

### The questionnaire

Data collection used the Cultural Competence Assessment Tool (CCATool), which is based on Papadopoulos, Tilki, and Taylor’s 1998 model of cultural competence [20]. Permission to use the original CCATool questionnaire for research purposes was obtained from the copyright holder, Irene Papadopoulos. The original questionnaire has demonstrated high reliability with a Cronbach’s alpha coefficient greater than 0.7 [21]. The CCATool includes four subscales: cultural awareness, cultural knowledge, cultural sensitivity, and cultural skills. Each subscale of the questionnaire included ten statements that participants rated on a 4-point Likert scale. The scale contained the following response options: 5 - strongly agree, 4 - agree, 2 - disagree, and 1 - disagree at all. The scale did not include a neutral option, as it aimed to capture participants’ agreement or disagreement with each statement. To facilitate self-assessment of cultural awareness, knowledge, sensitivity, and skills, each section also included a visual analogue scale (VAS). Four statements within the cultural sensitivity subscale were reverse coded, meaning that higher scores indicated lower levels of cultural sensitivity. In each subscale, participants could achieve a maximum score of 10 points and a minimum score of 0 points. A perfect score of ten in all four subscales was considered indicative of cultural competence and meant that students possessed a high level of cultural competence. The CCATool can be used for self-assessment by health professionals as well as for organizational assessment to identify strengths and areas for improvement in cultural competence. It provides valuable insight into individual or collective readiness to provide culturally congruent care and helps guide targeted interventions and training programs to improve cultural competence [9].

To ensure linguistic accuracy and cross-cultural validity, a translation process was used for the questionnaire. First, the questionnaire was translated from English into Slovenian. The back-translation method described by Iliescu [22] was used. Two experts, knowledgeable in the field and experienced in quantitative research methods, worked together to produce the translated version. They carefully reviewed the translation to ensure terminological consistency. Subsequently, the Slovenian version of the questionnaire was translated back into English and formed the basis for the comparison. Both versions of the questionnaire underwent a thorough validation process, assessing the validity and clarity of the questionnaire. As part of a pilot study, the questionnaire was administered to a sample of 10 university faculty members in the fields of sociology, nursing, and transcultural nursing. The aim was to assess the comprehensibility and face validity of the questionnaire. The experts involved in the pilot study confirmed that all items of the questionnaire were clear, concise and easy to understand. In addition, the internal consistency of each of the four subscales was assessed, yielding an acceptable level of reliability. The internal consistency of the individual subscales was satisfactory, as evidenced by Cronbach’s alpha coefficients between 0.79 and 0.85 [23].

### Data collection

The questionnaire was distributed to students via email in the form of an online survey by the researchers. Participation in the survey was voluntary, and students gave their consent by clicking on the embedded link, indicating their agreement to participate, and starting the survey. The online questionnaire, which was administered via the open source application EnKlikAnketa (www.1ka.si), could be completed throughout the month of May 2022. The questionnaire included a comprehensive explanation of the purpose of the survey and instructions on how to complete it. To maintain anonymity, a database was set up to store participants’ responses without personal information. Only the lead researcher had access to the data during the analysis phase.

### Data analysis

Data obtained from EnKlikAnketa (www.1ka.si) were exported for statistical analysis using IBM SPSS version 26.0 (SPSS Inc, Chicago, IL, USA). Statistical methods used in this study included basic descriptive statistics such as frequency, minimum, maximum, mean, median, and standard deviation. In addition, inferential statistics were applied, particularly the chi-square test of independence. Given the non-normal distribution of the data (confirmed by the Shapiro-Wilk test, p < 0.05), non-parametric tests were used to determine statistically significant differences between groups. The Mann Whitney U test and the Kruskal-Wallis H test were used for group comparisons, while the Wilcoxon signed-rank test was used to compare two sets of results from the same participants. A p-value of ≤ 0.05 was considered the threshold for statistical significance.

## Results

### Participants demographics

Table 1 shows that the majority of students were female. The age of the students ranged from 18 to 33 years (M*dn* = 22.5; SD = 3.2). The sample was balanced in terms of students’ place of residence, with an even distribution between urban and rural areas. More than half of the students identified themselves as religious. While most students had not lived in a country other than their country of birth, it is notable that 68% of students had relatives abroad. Regarding interactions with people of other ethnic backgrounds, 75% of students reported having such interactions more frequently, while 25% reported very infrequent interactions, occurring either once a week or month or a few times a year.

**Table 1:** Demographic characteristics of participants (n = 180)

Variables	n	%
Gender		
Male Female	34 146	18.9 81.1
Country of birth		
Slovenia Other (Bosnia and Herzegovina, Croatia, Italy, Kosovo, Macedonia, Moldova, Serbia)	150 30	83.3 16.7
Have you lived in a foreign country for more than 6 months?		
Yes No	33 147	18.4 81.6
Do you have relatives living in a foreign country? Yes No	124 56	68.9 31.1
How would you define your religious beliefs?		
I am religious. I am an atheist. I am an agnostic. I do not wish to answer.	108 43 8 21	60.0 23.9 4.4 11.7
Place of residence		
City A suburb The countryside	80 24 76	44.4 13.4 42.2
To what extent do you meet people from other ethnic groups?		
Daily Several times a week Once a week Once a month A few times a year	81 54 20 13 12	45.0 30.0 11.1 7.3 6.6

### Level of cultural competence

The cumulative score obtained using CCATool coding was high and above the calculated median for the CCATool (M*dn* = 32.0, SD = 7.9; 95% confidence interval [29.8, 31.6], p < 0.01), as shown in Table 2.

**Table 2:** Descriptive statistics of cultural competence assessment

Variable	n	M*dn*(IQR)	M±SD	95% CI		*p* value
				Lower	Upper	
Cultural awareness	10	9.00(2)	8.75±1.615	8.50	8.99	< 0.001
Cultural knowledge	10	7.00(3)	7.27±1.726	6.95	7.59	< 0.001
Cultural sensitivity	10	6.00(2)	6.01±1.986	5.65	6.37	< 0.001
Cultural skills	10	10.00(2)	8.68±1.873	8.34	9.02	< 0.001
CCATool	40	32.00(29)	30.70±7.918	29.78	31.63	< 0.001

Note. n − number of statements; M − Mean; SD − Standard Deviation; M*dn* − Median; IQR − Interquartile Range; 95% CI − 95% Confidence Interval

The findings indicate that the respondents display a high level of cultural competence. Moreover, the results reveal strong cultural awareness among the participants. Additionally, the respondents demonstrated proficiency by providing correct answers on nine out of ten statements. However, there is scope for improvement in cultural sensitivity, which received the lowest scores. Although a perfect score of ten on all four subscales indicates comprehensive cultural competence, the students scored 32 out of 40 based on CCATool coding.

Each of the four subscales contained one or two statements that respondents self-rated on a visual analogue scale (VAS) ranging from 1 (lowest/negative) to 10 (highest/positive). The purpose of these statements was to compare students’ self-assessment based on VAS with their final score on each subscale and to identify possible discrepancies (see Table 3).

**Table 3:** Comparison between the VAS self-assessed scores and objective scores resulting from the coded statements on each subscale - Wilcoxon Signed-Rank Test

Variable	M±SD	M*dn*(IQR)	Wilcoxon Signed Ranks test	
			Z	*p*
I am highly aware of my own ethnic and cultural identity (VAS self-assessed question).	7.77±2.064	8(4)	‒4.797	< 0.001
*Cultural awareness subscale*	8.75±1.615	9.00(2)		
I am very well informed about the culture and social situation of the majority of my clients (VAS self-assessed question).	5.78±2.018	6(2)	‒5.747	< 0.001
*Cultural knowledge subscale*	7.27±1.726	7.00(3)		
I am very comfortable working with people whose beliefs, values and practices are different from my own (VAS self-assessed question).	6.81±2.342	7(4)	‒3.082^b^	0.002
I am very confident of my ability to establish trust, show respect and empathy to all people whatever their culture.	8.36±1.745	9(3)	‒7.082^b^	< 0.001
*Cultural sensitivity subscale*	6.01±1.986	6.00(2)		
I am very able to incorporate the clients cultural beliefs into the care and treatment I provide (VAS self-assessed question).	7.53±1.842	8(2)	‒5.179	< 0.001
I am very confident to challenge racism and discrimination towards clients, carers and staff (VAS self-assessed question).	7.51±2.1622	8(4)	‒4.909	< 0.001
*Cultural skills subscale*	8.68±1.873	10.00(2)		

Note. M − Mean; SD − Standard Deviation; M*dn* − Median; IQR − Interquartile Range; ^b^− Based on positive ranks

The results showed a remarkable discrepancy between the subjective perception of cultural awareness and its objective measurement, as participants scored higher on the rated statements than on the self-assessed statement (p < 0.001) using VAS. Similar results were observed for the subscales assessing cultural knowledge and cultural skills. In both cases, students scored higher on the rated statements within each subscale than on the self-assessed statements (p = 0.002 and p < 0.001, respectively) using VAS. However, for the subscale cultural sensitivity, the self-assessed values on the (VAS) were significantly higher than the values resulting from the statements assessed by the students on the scale (p < 0.001).

### Factors associating cultural competence among nursing students

To identify any statistically significant differences between the groups, the Mann-Whitney U-test, the Kruskal-Wallis H-test and the chi-square test for independence were conducted (Table 4).

**Table 4:** Comparison of the scores of the CCATool and its subscales based on student characteristics

Variables	Awareness M±SD M*dn*(IQR)	Knowledge M±SD M*dn*(IQR)	Sensitivity M±SD M*dn*(IQR)	Skills M±SD M*dn*(IQR)	CCATool M±SD M*dn*(IQR)
Gender					
Male	8.41±2.239 9.00(2)	6.59±1.709 7.00(3)	5.95±1.914 6.00(2)	8.27±2.567 9.00(3)	29.23±6.406 30.00(7)
Female	8.82±1.123 9.00(2)	7.42±1.765 7.00(3)	6.02±2.035 6.00(2)	8.77±1.687 10.00(2)	31.03±4.846 32.00(5)
***U test***	1351.000	1002.500	1126.000	1017.000	1282.000
***p value***	> 0.05	> 0.05	> 0.05	> 0.05	> 0.05
Place of birth					
Born in Slovenia	8.85±1.138 9.00(2)	7.19±1.733 9.00(2)	5.92±1.968 6.00(2)	8.70±1.693 9.00(2)	30.66±4.812 31.0(6)
Born outside Slovenia	8.17±2.358 9.00(2)	7.78±2.045 8.00(4)	6.28±2.137 6.00(2)	8.44±2.770 10.00(2)	30.67±7.187 32.50(11)
***U test***	1011.000	1002.000	1024.000	908.500	1249.500
***p value***	> 0.05	< 0.05	> 0.05	> 0.05	> 0.05
Religious beliefs					
Religious students	8.79±1.192 9.00(2)	7.37±1.680 7.00(3)	6.25±2.021 6.00(2)	8.76±1.735 9.50(2)	31.17±4.862 32.00(5)
Atheist students	8.77±1.832 9.00(2)	7.57±1.906 7.00(2)	5.53±1.995 5.00(2)	8.50±2.301 10.00(3)	30.37±5.968 31.50(6)
***U test***	1527.500	1287.000	996.000	1124.000	1499.000
***p value***	> 0.05	> 0.05	> 0.05	> 0.05	> 0.05
Meeting people from other ethnic groups					
Daily	8.66±1.283 9.00(2)	7.05±1.645 7.00(2)	5.84±2.078 6.00(3)	8.64±1.773 10.00(3)	30.20±5.047 31.00(7)
Several times a week	9.07±0.829 9.00(2)	7.33±1.886 7.00(3)	5.95±2.136 6.00(2)	8.70±1.911 9.50(2)	31.05±5.129 32.00(6)
Once a week	8.70±1.567 9.50(3)	7.90±1.853 8.50(3)	6.70±1.889 6.50(3)	9.20±1.135 10.00(2)	32.50±18.722 33.00(7)
Once a month	9.99±1.069 9.00(2)	8.00±2.000 8.50(3)	6.25±1.389 6.00(2)	9.25±1.165 10.00(2)	32.50±4.106 32.50(14)
A few times a year	8.57±0.787 8.00(1)	7.29±1.799 7.00(1)	6.43±1.718 7.00(3)	8.71±1.113 9.00(2)	31.00±3.317 30.00(6)
***χ2***	4.831	2.472	1.781	1.575	3.258
***p value***	> 0.05	> 0.05	> 0.05	> 0.05	> 0.05

Note. M − Mean; SD − Standard Deviation; M*dn* − Median; IQR − Interquartile Range; U test − Mann Whitney test; χ2 − Kruskal-Wallis test

The results revealed intriguing patterns in relation to several factors that associate the level of cultural competence measured by the CCATool. Although there were some differences in performance between female and male students, these differences did not reach statistical significance (p > 0.05). Similarly, students born outside Slovenia, including Bosnia and Herzegovina, Croatia, Italy, Kosovo, Macedonia, Moldova and Serbia, showed higher levels of cultural competence; however, these differences were not statistically significant (p > 0.05). Nevertheless, statistically significant differences (p < 0.05) were found in the Cultural Knowledge subscale between students born in Slovenia and those born outside Slovenia. In addition, students who described themselves as atheists showed higher levels of cultural competence than their religious peers, but again no statistically significant differences were found (p > 0.05). Interestingly, students who lived in suburban or rural areas showed higher levels of cultural competence than students who lived in urban areas (Mdn = 31.00, SD = 7.412; Mdn = 31.00, SD = 8.207 and Mdn = 30.00, SD = 7.873 respectively), although these differences were not statistically significant (p > 0.05). In addition, the chi-square test for independence was conducted to examine the relationship between the level of cultural competence and a stay abroad of more than 6 months. The results showed a statistically significant relationship (χ(2) = 50.550, p = 0.020) between these two variables, indicating that a stay abroad of more than 6 months is associated with higher levels of cultural competence. However, when examining the relationship between the level of cultural competence and the presence of parents born in another country or relatives living abroad, there was no statistically significant relationship (p > 0.05) between these variables.

## Discussion

Establishing a transcultural nursing curriculum in health education, which is predominantly biomedically driven and rarely encourages enough self-reflection on social and cultural curricular content and context of care despite advocating for an inclusive approach, presents a challenge in many educational settings worldwide [24]. In this study, we examined the self-assessed levels of cultural competence in undergraduate nursing students. This was done a decade after the first changes towards a more transculturally oriented nursing curriculum were introduced at UP FHS. Tracking the impact of curricular modifications on the development of cultural competence in future healthcare professionals in a rapidly changing social and cultural context of care has a significant bearing on future changes to the nursing curriculum.

Our study indicates that undergraduate nursing students demonstrated a positive development in their cultural competence, as they score relatively high overall on the cultural competence assessment. Consistent with previous research on the cultural competence of nursing students, it was found that individuals who receive more culturally related education show higher levels of cultural competence and that the latter increases with students’ grade level, and that teachers show higher levels of cultural competence than students [17, 18]. A more detailed analysis of the cultural competence assessment subscales revealed that nursing students performed exceptionally well in the areas of cultural awareness and cultural skills. Cultural awareness involves the conscious recognition of one’s own cultural background and helps to avoid stereotyping and prejudice towards other cultural groups. Cultural competence, on the other hand, refers to the ability to obtain necessary information from patients through culturally appropriate health assessments [6]. The higher performance in these subscales may be attributed to the nursing students’ exposure to a multicultural and multilingual environment. The location of UP FHS in a bilingual area and the proximity to one of the largest ports in this part of the Adriatic probably contribute to this experience [19]. In addition to the elements of transcultural care already implemented in the nursing curriculum, supervised and guided clinical practice can provide an additional cultural immersion experience and contribute to a greater development of cultural competences when caring for patients from diverse cultural backgrounds [13, 18]. Moreover, in their first year of study, nursing students’ clinical practice, where the basis for a culturally appropriate health assessment and skill development is set, is mainly supervised by university-employed clinical mentors. In the second and third years of study, clinical mentors are mainly nurses employed at healthcare institutions, and university teachers only assume the role of practice supervisors and coordinators. Compared to a similar study conducted at UP FHS in 2018 [17], this study found that nursing students’ levels of cultural awareness and clinical skills were higher. On the other two subscales, “Cultural Knowledge” and “Cultural Sensitivity” which focused mainly on the development of appropriate communication skills, scores were low, indicating students’ relatively low level of cultural competence. Similar to Wang et al. [19], we can also assume that the main reason for lower scores on these two subscales may be students limited direct exposure to transcultural nursing content in the undergraduate nursing curriculum. The currently available hours for theoretical content (less than 5 h in a three-year nursing programme) are definitely not sufficient to achieve a desirable level of cultural competence, especially if we wish to stimulate its development also after graduation. As a consequence of the limited number of hours in the curriculum, the focus on culturally sensitive communication was also very limited. From this perspective, future nursing curriculum should focus on transcultural components and rely on cross-curricular integration. Cross-curricular integration is a holistic didactic approach that characterises both horizontal and vertical integration of knowledge, content and learning skills, and encourages an independent and active acquisition of learning experiences. Such linking of disciplines builds on modern theories of teaching and learning and puts learners in the role of active constructors of their own knowledge. This can happen either at the content, conceptual or process level [25].

Cultural competence is an essential skill that nurses must possess in order to provide culturally congruent care. The results of our study show that undergraduate nursing students have made positive progress in their cultural competence, as indicated by their relatively high overall scores on the cultural competence assessment. By promoting cultural competence in nursing students, we can better enable them to provide patient-centred care that recognises and respects each individual’s cultural background and values. This in turn will contribute to more effective and inclusive healthcare [26]. It is critical to continue to monitor and evaluate the impact of curriculum changes on the development of cultural competence. This ongoing evaluation will inform future updates to nursing curricula and ultimately shape the future work environment and ensure high quality, culturally sensitive care [27]. As nursing students develop their cultural competence, they will be better able to improve the health and overall well-being of patients from diverse cultural backgrounds, which is consistent with the fundamental goal of culturally congruent care [28].

It is important to acknowledge some limitations of this study that should be considered when interpreting its results. First, data collection was based on a self-completed questionnaire, which may lead to bias or limited accuracy of responses [29]. In addition, the study focused on a specific cohort of nursing students from a single faculty, primarily due to newly introduced changes in the nursing curriculum in Slovenia. To gain a comprehensive understanding of the development of cultural competence, future research should include a nationally representative sample of nursing students to enable comparisons and facilitate the formulation of curriculum changes or policies on a broader level. Longitudinal and experimental studies would provide valuable insights into the development of cultural competence. For future work, a test-retest study design should be considered to assess changes more accurately in cultural competence over time. In addition, conducting international comparative studies in countries with similar nursing education and curriculum structures would serve as a benchmarking tool and ensure the pursuit of quality in higher education.

## Conclusion

This study sheds light on the cultural competence of nursing students in Slovenia. It reveals a general strength in this area, but also shows room for targeted educational strategies, especially in cultural sensitivity. A divergence was also found between the students’ self-assessed cultural competence and the objective scores measured by the CCATool. Furthermore, the study shows differences in cultural competence that are influenced by demographic and social variables, such as the length of time spent living abroad. Although the study does not measure changes in competence levels over time, its findings serve as an important reference for decision-makers in education and health policy. The data support the need for ongoing evaluations, curriculum updates that integrate diverse cultural perspectives, and a commitment to promoting ongoing education in cultural competence to equip nursing students for effective practise after graduation.

### Relevance to clinical practice

Increasing cultural diversity among healthcare users poses new demands and challenges for healthcare professionals, especially nursing students, who will be working in dynamic and globally connected work environments in the future. Understanding and developing cultural competences are key factors in successfully addressing the diverse needs of patients and building trusting relationships between patients and health professionals. Understanding the development of cultural competences and their importance to the future nursing work environment has important implications for education programmes and policies related to health. A comprehensive and well-rounded education that includes the acquisition of cultural competences will enable future healthcare professionals to work effectively and efficiently in a diverse environment and improve the quality of patient care.

## Data Availability

The datasets generated and/or analysed during the current study are not publicly available due institutional data sharing clause but are available from the corresponding author on reasonable request.

## References

[1] International Organization for Migration (2022). World migration report 2022.

[2] Osmancevic S, Schoberer D, Lohrmann C, Großschädl F (2021). Psychometric properties of instruments used to measure the cultural competence of nurses: a systematic review. Int J Nurs Stud.

[3] Belintxon M, Carvajal A, Pumar-Méndez MJ, Rayon-Valpuesta E, Velasco TR, Belintxon U (2021). A valid and reliable scale to assess cultural sensibility in nursing. Nurse Educ Today.

[4] Sharifi N, Adib-Hajbaghery M, Najafi M (2019). Cultural competence in nursing: a concept analysis. Int J Nurs Stud.

[5] Prosen M (2015). Introducing transcultural nursing education: implementation of transcultural nursing in the postgraduate nursing curriculum. Procedia - Soc Behav Sci.

[6] Tosun B, Yava A, Dirgar E, Şahin EB, Yılmaz EB, Papp K (2021). Addressing the effects of transcultural nursing education on nursing students’ cultural competence: a systematic review. Nurse Educ Pract.

[7] Oikarainen A, Mikkonen K, Kenny A, Tomietto M, Tuomikoski A-M, Meriläinen M (2019). Educational interventions designed to develop nurses’ cultural competence: a systematic review. Int J Nurs Stud.

[8] Prosen M (2018). Developing cross-cultural competences. Obz Zdr Nege.

[9] Papadopoulos I. Transcultural Health and Social Care: development of culturally competent practitioners. 1st ed. Churchill Livingstone; 2006.

[10] Papadopoulos I (2018). Culturally competent Compassion: a guide for Healthcare students and practitioners.

[11] Antón-Solanas I, Tambo-Lizalde E, Hamam-Alcober N, Vanceulebroeck V, Dehaes S, Kalkan I (2021). Nursing students’ experience of learning cultural competence. PLoS ONE.

[12] Osmancevic S, Großschädl F, Lohrmann C (2023). Cultural competence among nursing students and nurses working in acute care settings: a cross-sectional study. BMC Health Serv Res.

[13] Dobrowolska B, Gutysz-Wojnicka A, Ozga D, Barkestad E, Benbenishty J, Breznik K (2020). European intensive care nurses’ cultural competency: an international cross-sectional survey. Intensive Crit Care Nurs.

[14] Nuri-Robins K, Lindsey DB, Lindsey RB, Terrell RD (2011). Culturally proficient instruction: a guide for people who teach.

[15] Hughes V, Delva S, Nkimbeng M, Spaulding E, Turkson-Ocran R-A, Cudjoe J (2020). Not missing the opportunity: strategies to promote cultural humility among future nursing faculty. J Prof Nurs.

[16] Greene-Moton E, Minkler M (2020). Cultural competence or Cultural Humility? Moving beyond the debate. Health Promot Pract.

[17] Ličen S, Karnjuš I, Prosen M (2021). Measuring Cultural Awareness among Slovene nursing student: a cross-sectional study. J Transcult Nurs.

[18] Lin H-L, Guo J-L, Chen H-J, Liao L-L, Chang L-C (2021). Cultural competence among pre-graduate nursing students, new graduate nurses, nurse mentors, and registered nurses: a comparative descriptive study. Nurse Educ Today.

[19] Wang Y, Xiao LD, Yan P, Wang Y, Yasheng A (2018). Nursing students’ cultural competence in caring for older people in a multicultural and developing region. Nurse Educ Today.

[20] Papadopoulos I, Lees S (2002). Developing culturally competent researchers. J Adv Nurs.

[21] Papadopoulos I, Tilki M, Lees S (2004). Promoting cultural competence in health care through a research-based intervention in the UK. Divers Health Soc Care.

[22] Iliescu D (2017). Adapting tests in linguistic and cultural situations.

[23] Field A. Discovering statistics using IBM SPSS statistics, 4th Edition. 4th ed. SAGE Publications Ltd; 2013.

[24] Klenner M, Mariño R, Pineda P, Espinoza G, Zaror C (2022). Cultural competence in the nursing, dentistry, and medicine professional curricula: a qualitative review. BMC Med Educ.

[25] Volk M, Štemberger T, Sila A, Kovač N (2020). Cross-curricular integration: the path to the realisation of Educational Goals.

[26] Rahimi M, Khodabandeh Shahraki S, Fatehi F, Farokhzadian J (2023). A virtual training program for improving cultural competence among academic nurse educators. BMC Med Educ.

[27] Kaihlanen A-M, Hietapakka L, Heponiemi T (2019). Increasing cultural awareness: qualitative study of nurses’ perceptions about cultural competence training. BMC Nurs.

[28] Oldland E, Botti M, Hutchinson AM, Redley B (2020). A framework of nurses’ responsibilities for quality healthcare — exploration of content validity. Collegian.

[29] Dillman DA, Smyth JD, Christian LM (2014). Internet, phone, mail, and mixed mode surveys: the tailored design method.

[30] World Medical Association. WMA Declaration of Helsinki: Ethical Principles for Medical Research Involving Human Subjects. 2018. https://www.wma.net/policies-post/wma-declaration-of-helsinki-ethical-principles-for-medical-research-involving-human-subjects/. Accessed 8 Dec 2021.

